# Coronary Stenosis Physiology and Novel Technologies

**DOI:** 10.5041/RMMJ.10398

**Published:** 2020-04-29

**Authors:** Nili Schamroth Pravda, Ran Kornowski

**Affiliations:** 1 Department of Cardiology, Rabin Medical Center, Petach Tikva, Israel; 2 The Faculty of Medicine, Tel Aviv University, Tel Aviv, Israel

**Keywords:** Coronary artery disease, FFR, FFRangio, iFR

## Abstract

An accurate functional assessment of coronary artery stenosis is pivotal in the management and clinical outcomes of patients. The hemodynamic relevance of coronary artery stenoses can be assessed using coronary flow surrogates, namely fractional flow reserve (FFR) and instantaneous wave-free ratio (iFR). This review provides an overview of these indexes, their clinical relevance, as well as a review of the literature supporting their use. It also reviews novel image-based FFR (e.g. FFRangio), the evidence showing the accuracy of this technique when compared to conventional wire-based techniques, as well as the clinical implications of non-invasive coronary artery stenosis functional assessments.

## INTRODUCTION

Coronary artery disease (CAD) is one of the leading causes of morbidity and mortality worldwide.^1^ An accurate diagnostic assessment of CAD is pivotal in the management and clinical outcomes of patients. Both quantitative and hemodynamic assessments of coronary stenoses are vital in the prognostic stratification and management of patients with CAD. Functional assessment of CAD can be carried out using stress or resting indexes, namely fractional flow reserve (FFR) and instantaneous wave-free ratio (iFR). These indexes are surrogate markers of coronary flow and assess the functional significance of coronary stenosis to help guide percutaneous coronary intervention.

## WIRE-BASED TECHNIQUES (FFR/IFR)

Fractional flow reserve (FFR) is a wire-based technique used to measure pressure differences across a coronary stenosis and thus assess its hemodynamic relevance. It is calculated as the maximum myocardial blood flow in a stenotic territory, divided by normal maximum blood flow in that same territory.^2^ It is obtained by measuring the ratio of distal coronary pressure to the aortic pressure using pressure-measuring guidewires during pharmacologically induced maximal coronary artery vasodilation. It is generally accepted that a stenosis with a FFR value of less than 0.8 is physiologically significant and thus the lesion should be revascularized. Lesions with a FFR above 0.8 are regarded as physiologically non-ischemic and can be treated medically using pharmacotherapy and/or lifestyle change recommendations.

Three landmark studies have led the evolution of FFR-guided revascularization, namely the DEFER, FAME, and FAME 2 clinical trials.^3^–^5^

The DEFER (Deferral versus Performance of Percutaneous Transluminal Coronary Angioplasty in Patients Without Documented Ischaemia)^3^ trial enrolled 325 patients with stable chest pain scheduled for percutaneous coronary intervention (PCI) of an intermediate stenosis defined as an angiographic stenosis of >50% diameter. Its aim was to assess the outcome and safety of deferring PCI in angiographic stenoses with FFR>0.75. Patients were randomly assigned to deferral (*n*=91) or performance of PCI (*n*=90) if FFR was <0.75. At 1-year follow-up, event-free survival rates were similar in the deferral and FFR-guided PCI groups (89% versus 83%, respectively; *P*=0.27).^3^ A 5-year follow-up of DEFER patients showed that these outcomes were maintained over time.^6^ Event-free survival did not differ between the “Defer” and “Perform” groups (80% and 73%, respectively; *P*=0.52). The composite rate of cardiac death and acute myocardial infarction (AMI) in the “Defer” and “Perform” groups was not significantly different (3.3% versus 7.9%; *P*=0.21). These results indicate that many patients referred for PCI on the basis of an angiographic significant coronary stenosis have functionally non-significant lesions as indicated by their FFR index.

The FAME trial (Fractional Flow Reserve versus Angiography for Multivessel Evaluation)^4^ compared two different revascularization strategies: FFR-guided PCI (revascularization of lesions with an FFR ≤0.80) compared to angiography-guided PCI (revascularization of lesions with >50% stenosis) in 1,005 patients with stable coronary artery disease and multivessel disease.^4^ Results showed that at the 1-year follow-up the primary endpoint (a composite of death, myocardial infarction [MI], and repeat revascularization) was significantly reduced in the FFR-guided group compared with the angiographic-guided group (13.2% versus 18.3%; *P*=0.02). Further benefit of the FFR strategy was a decrease in the number of stents and the amount of contrast used. Of note, 65% of those with an angiographic stenosis of 50%–70% did not have hemodynamically significant lesions, nor did 20% of those with a stenosis of 71%–90%. This highlights the poor correlation between diameter stenosis by visual estimation versus functional relevance. Long-term follow-up at 5 years showed that major adverse cardiac events (MACE) occurred in 31% of patients in the angiography-guided group versus 28% in the FFR-guided group (relative risk 0.91; 95% CI 0.75–1.10; *P*=0.31).^7^ This study showed that FFR-guided PCI can lead to improved patient outcomes and prevent unnecessary stenting.

The FAME 2 trial (Fractional Flow Reserve versus Angiography for Multivessel Evaluation 2) investigated the outcomes of 1,220 patients with stable angina and at least one stenosis with FFR ≤0.80, in which it compared those receiving FFR-guided PCI with patients who received optimal medical therapy alone.^5^ Recruitment was stopped early due to the compelling results showing that PCI using drug-eluting stent (DES) implantation decreased the primary endpoint of death, non-fatal MI, or urgent revascularization within 2 years compared with medical treatment alone (4.3% versus 12.7%; *P*<0.001). This was driven by a significantly lower need for urgent revascularization (1.6% versus 11.1%; hazard ratio [HR] 0.13; 95% CI 0.06–0.30; *P*<0.001). The benefit of FFR-guided PCI over medical therapy alone was still significant at 3 years’ follow-up (MACE 10.1% versus 22.0%; *P*<0.001).^8^ The conclusion was that hemodynamically significant lesions should be revascularized rather than treated with optimal medical therapy.

Based on the above evidence, the 2018 European Society of Cardiology (ESC) Guidelines on Myocardial Revascularization regards coronary pressure-derived FFR as the standard of care for the functional assessment of lesion severity in patients with intermediate-grade stenosis without evidence of ischemia in non-invasive testing (Class I recommendation based on Level of Evidence A), and in those with multivessel disease (Class IIa Recommendation based on Level of Evidence B).^9^

Another wire-based physiological index that does not require pharmacologic hyperemia is iFR. This index is based on the diastolic wave-free period. This is a time frame in the cardiac cycle during which resistance at rest is stable. Therefore, at this time the coronary flow is proportional to the ratio of the proximal and distal coronary artery pressures.^2^ An iFR of ≤0.89 has been found to be comparable to FFR of ≤0.80.

In the DEFINE-FLAIR trial, 2,492 patients with coronary artery disease were randomized to undergo either iFR-guided or FFR-guided coronary revascularization. The primary endpoint of MACE (composite of death from any cause, non-fatal MI, or unplanned revascularization) at 1 year occurred in 6.8% in patients randomized to iFR-guided revascularization versus 7.0% in patients randomized to FFR-guided revascularization (*P*<0.001 for non-inferiority; HR 0.95; 95% CI 0.68–1.33; *P*=0.78).^10^ Coronary revascularization guided by iFR was non-inferior to revascularization guided by FFR.

In the iFR-SWEDEHEART trial, 2,037 participants with stable angina or an acute coronary syndrome were randomly assigned to undergo revascularization guided by either iFR or FFR; iFR was non-inferior to FFR, with the primary endpoint of death from any cause, non-fatal MI, or unplanned revascularization occurring in 6.7% of the iFR group and 6.1% in the FFR group (*P*=0.007 for non-inferiority; HR 1.12; 95% CI 0.79–1.58; *P*=0.53).^11^

## IMAGE-BASED FFR: THE UNMET NEED

The wired-based FFR (wbFFR) technique has some limitations despite robust evidence to justify its use during coronary angiography assessment. These shortcomings include the need for an invasive procedure utilizing a designated pressure-wire as well as the need for pharmacological vasodilation, its time-consuming nature, and the need for highly skilled operators. In an era of advancing technology, angiographic image-based FFR has the potential to offer an image-based, less invasive solution.

**Figure 1:**
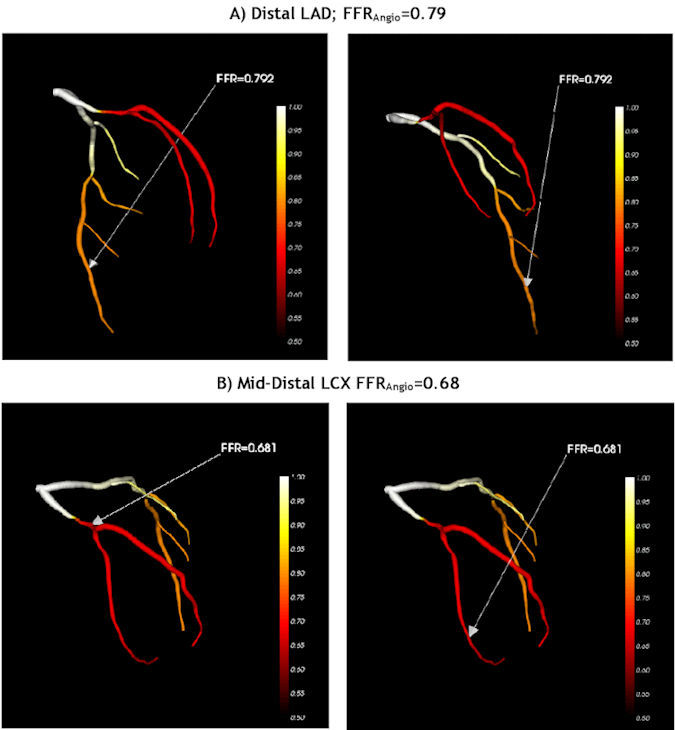
FFR-Angiography Images of a Patient with Coronary Artery Disease Vessel physiology is illustrated by color-coded imaging using the CathWorks technology. **A:** left anterior descending coronary artery (LAD; FFRangio=0.79, functionally significant stenosis but in the “grey zone”). **B:** left circumflex (LCX; FFRangio=0.68, functionally significant stenosis).

FFRangio™ (CathWorks, Kfar-Saba, Israel) is an innovative technology solution providing a functional angiographic three-dimensional mapping of the full coronary vasculature. It is based on a lumped-element model that can assess FFR using routine angiograms and the dynamic characteristics of the vessel as well as the subject’s hemodynamic information.^9^–^12^ Within few minutes of automatic processing, a non-invasive measurement of FFR can be provided and depicted on the angiogram screen (Figure 1).

Following successful validation in several human trials, this technology has been found to be highly reproducible, and the diagnostic accuracy of FFRangio is comparable to that of wbFFR.^12^–^15^ One of the pivotal trials in this field is the FAST-FFR trial.^16^ The FAST-FFR (FFR_angio_ Accuracy versus Standard FFR) trial was a multinational prospective trial of 301 patients with 319 vessel assessments. The per-vessel sensitivity and specificity of FFRangio compared with conventional wbFFR was 94% (95% CI 88%–97%) and 91% (95% CI 86%–95%). The FFRangio values correlated well with FFR measurements (*r*=0.80; *P*<0.001) in a wide range of coronary lesion severity and physiology assessment. This technology has also been tested in patients with multivessel disease, with similar findings, including an excellent accuracy of 92.3% (95% CI 79.1%–98.4%) of FFRangio compared to wbFFR, with Pearson’s *r* between wbFFR and FFRangio being 0.83.^13^ Furthermore, this study also found that FFRangio measurement was performed significantly faster than wbFFR. These results highlight the potential of FFRangio as a tool that can help further the implementation of functional assessment of coronary lesions in routine clinical practice.

Another pooled-analysis of five prospective cohort studies reported similar findings, demonstrating good diagnostic performance of FFRangio.^17^ The diagnostic accuracy of FFRangio was 93%, the mean difference between wbFFR and FFRangio was 0.00± 0.12, and the correlation coefficient was 0.83 (*P*<0.001). These results were consistent across all patient and lesion subgroups. This further solidifies the diagnostic abilities of this mode of coronary physiology assessment.

## ADDITIONAL ANGIOGRAPHY-BASED SOLUTIONS

Additional computational techniques have been developed in order to address the shortfalls and challenges of angiography-based FFR analysis. These techniques are based on rapid angiographic flow analysis, computational fluid dynamics, and/or mathematical formulas.^18^–^20^ Similar to FFRangio, these techniques have shown good correlation with the wbFFR but have limited capacity to provide the functional assessment of the full coronary tree in real time, i.e. “online.” Nonetheless, a recent systematic review and meta-analysis has examined the performance of the various angiography image-based FFR techniques pooled together and shows very good correlation with the invasive FFR approach.^21^ This analysis included 13 studies comprising 1,842 vessels. The results showed that angiographically derived FFR, regardless of the technology being utilized, was accurate to detect hemodynamically significant coronary lesions when compared to wbFFR as a reference. A Bayesian bivariate meta-analysis showed a pooled sensitivity of 89% (95% credible interval 83%–94%) and specificity of 90% (95% credible interval 88%–92%), with a summary area under the receiver-operating curve of 0.84 (95% credible interval 0.66–0.94).^21^

## CLINICAL IMPLICATIONS

The clinical implications of an angiography-derived FFR is that a functional assessment of coronary lesions can be done accurately, quickly, and in a non-invasive manner. This technology could make the hemodynamic assessment of coronary artery stenoses cheaper and more accessible, and thereby has the potential to increase the implementation of hemodynamic assessment of coronary artery stenoses.

In summary, the functional assessment of CAD is pivotal for an accurate assessment of coronary artery stenosis and the management decisions that follow. The literature highlights the value and supports the use of coronary flow surrogates for this purpose. Indeed, FFR has become the clinical standard for assessing the hemodynamic functional relevance of coronary artery lesions. There is an increasing body of evidence showing the accuracy of non-invasive angiographically derived FFR when compared to wbFFR. This has been validated predominantly in studies of relatively non-complex lesions. Further validation is needed to support its use in more complex lesions such as diffuse coronary disease, serial lesions and bifurcation lesions, and in left main stem disease in which the use of wbFFR is also limited.

## Abbreviations

AMI: acute myocardial infarction
CAD: coronary artery disease
DES: drug-eluting stent
ESC: European Society of Cardiology
FFR: fractional flow reserve
iFR: instantaneous wave-free ratio
MACE: major adverse cardiac events
PCI: percutaneous coronary intervention
wbFFR: wired-based fractional flow reserve
